# Superior Orbicularis Oris Muscle Activity in Children Surgically Treated for Bilateral Complete Cleft Lip and Palate

**DOI:** 10.3390/jcm10081720

**Published:** 2021-04-16

**Authors:** Liliana Szyszka-Sommerfeld, Monika Elżbieta Machoy, Sławomir Wilczyński, Mariusz Lipski, Krzysztof Woźniak

**Affiliations:** 1 Department of Orthodontics, Pomeranian Medical University in Szczecin, Al. Powst. Wlkp. 72, 70111 Szczecin, Poland; monika.machoy@pum.edu.pl (M.E.M.); krzysztof.wozniak@pum.edu.pl (K.W.); 2 Department of Basic Biomedical Science, Medical University of Silesia, Katowice, 3 Kasztanowa Street, 41200 Sosnowiec, Poland; swilczynski@sum.edu.pl; 3 Department of Preclinical Conservative Dentistry and Preclinical Endodontics, Pomeranian Medical University in Szczecin, Al. Powst. Wlkp. 72, 70111 Szczecin, Poland; lipam@pum.edu.pl

**Keywords:** bilateral cleft, cleft lip and palate, dentofacial deformities, facial growth, multidisciplinary cleft treatment, surgical lip repair, superior orbicularis oris muscle, surface electromyography

## Abstract

The aim of this cross-sectional study was to evaluate the electromyographic activity of the superior orbicularis oris muscle both in children surgically treated for bilateral complete cleft lip and palate (BCCLP) as well as in subjects without BCCLP. The study comprised 77 children aged 6.6 to 12.5 years. All the patients with clefts had previously undergone lip and palate surgery. The upper lip electromyographic (EMG) assessments were made with a DAB-Bluetooth device (Zebris Medical GmbH, Germany) at rest, while swallowing saliva, protruding lips and compressing lips. EMG measurements were also made when the subjects produced phonemes /p/, /b/, and /m/ with the vowel /a/. The Mann-Whitney U test was applied to statistically analyze the EMG values. Significantly higher median upper lip EMG activity under working conditions such as swallowing saliva, lip compression, and production of the phoneme /p/ with the vowel /a/ was observed in patients with BCCLP compared to those without a cleft. The results of the study showed that the upper lip muscle activity increases in children with BCCLP when swallowing saliva, compressing lips and during some speech movement tasks. This may be important in the aspect of the effect of surgical lip repair on the craniofacial growth.

## 1. Introduction

Clefts of the lip, alveolar process and/or palate are the most common congenital dentofacial abnormalities, which significantly affect the functions of the masticatory organ and disrupt the aesthetics of the face [1,2,3]. Effective treatment of a patient with cleft involves multidisciplinary surgical and non-surgical care from birth to adulthood [4,5]. The treatment outcome is affected by such factors as the extent of the morphological and functional changes involved, the type of cleft as well as the effectiveness of the primary surgery performed, including the surgeon’s skills, the choice of the surgical method, and the time and sequence of the surgical repair [6,7,8,9]. Surgical correction is the main premise of the current team approach to clefts management. A complete surgical design should effectively restore functions such as speech, chewing, breathing, hearing and facial appearance, while maintaining normal growth potential in the affected region [10,11,12]. 

Bilateral complete cleft lip and palate (BCCLP) is considered the most serious clinical form of cleft lip and palate (CLP) [13]. The primary manifestations of bilateral cleft lip are a procumbent or rotated premaxilla with a significantly increased width of the alar base and widely spaced lip segments, and very short columella [14,15,16]. Surgical treatment of BCCLP is the most difficult procedure among the common clefts of the lip and palate [17]. Long-term postoperative effects and the severity of age-related abnormalities pose a major challenge to clinicians dealing with the interdisciplinary treatment of patients with clefts including oral and maxillofacial surgeons and orthodontists. Major complications include hypoplasia of the maxilla, collapse of the maxillary buccal segments with a subsequent posterior crossbite, and premaxillary protrusion following a distorted facial profile [17,18].

Surgical lip repair is a very important procedure consisting in the immediate reconstruction of the cleft face and brings psychological benefits to the patient’s family [19]. Moreover, it should also be noted that lip repair may affect the final morphology of the maxilla and midface [20,21]. In 1977 Bardach and Eisbach forwarded the thesis that primary lip reconstruction always results in a certain degree of labial tension which is transferred as pressure to the underlying jaw. Recent studies have also shown that electromyographic (EMG) activity of the superior orbicularis oris muscle was higher following lip repair during swallowing saliva [22,23] and speech [24]. The authors suggested that the superior orbicularis oris muscle exerted a restrictive pressure effect on maxillary growth [22,23,24,25]. In unilateral complete CLP, this effect is usually negative because it excessively restricts maxillary anterior displacement [20,21,26,27,28,29,30]. However, the restraining lip repair effect may to some extent be desirable for facial growth in patients with bilateral CLP. In complete bilateral clefts, the increased backward pressure produced by the repaired orbicularis oris muscle is fundamental to the retropositioning of the protruded premaxilla, which results in an essential reduction in facial convexity [17,19,31,32]. 

It should also be noted that the orbicularis oris muscle has important functions in the stomatognathic system associated with food ingestion, facial expression or speech articulation [33] and abnormal lip activity during these functions may represent an additional factor threatening the integrity of underlying dentofacial structures. In light of the above, it is essential to determine the electrical activity of the upper orbicularis oris muscle during different functional conditions in subjects with CLP that have undergone cleft lip and palate surgery. Electromyography (EMG) is a tool that investigates muscle function by recording the electric signals coming from the muscles [34]. It makes it possible to assess the extent and duration of muscle activity. The use of surface electromyography (sEMG) significantly facilitates precise evaluation of muscle parameters [34,35,36]. Due to the simplicity of this method, its safety and availability, it has been used in research on children [37]. To date, a few of EMG studies of lip muscle function have been performed in patients with unilateral cleft lip and palate [23,24,38]. However, no reports have focused on the EMG activity of the superior orbicularis oris muscle in subjects with BCCLP.

The aim of this study was to determine whether the electrical activity of the upper orbicularis oris muscle at rest, during saliva swallowing, lip compression and during the production of the bilabial phonemes /p/, /b/ and /m/ associated with the vowel /a/ in children surgically treated for bilateral complete cleft lip and palate differs from that observed in children without a cleft. We hypothesized that there are no differences between the patients with and without BCCLP with regard to the EMG signals of the upper lip during these functional conditions.

## 2. Materials and Methods

The study was welcomed in accordance with the Helsinki Declaration, the protocol was approved by the Local Bioethics Committee of the Pomeranian Medical University (number KB-0012/08/15). Parental written informed consent was obtained for investigation of children before clinical and electromyographic procedures. Only consenting persons were included in the study.

A total of 185 subjects with mixed dentition were invited to participate in the study. Of them, five children’s parents did not express consent to voluntary participation their children in the research. One hundred and three children were excluded because they did not meet other inclusion criteria. The final sample comprised 77 children, divided into two groups: a cleft group and a noncleft group. 

The children with bilateral cleft were recruited from a total of 65 patients with clefts who were referred to the Cleft Treatment Centre in Szczecin in 2017 and underwent lip and palate surgical repair according to a similar operating protocol, which was as follows: a two-stage lip repair at the age of three to six months, followed by hard and soft palate closure in one operation at the age of approximately 12 months. Two-stage lip repair procedure started from the side of the wider cleft fissure. After six to eight weeks, the patients underwent lip closure on the opposite side. The triangular flap and rotation advancement Millard technique with primary elongation of nasal septum were used. The inclusion criteria for the cleft group were as follows: subjects of both sexes with mixed dentition, undergoing lip and palate closure, presence of a bilateral cleft lip and palate without a syndrome, a sequence, or karyotype abnormalities and consent to voluntary participation in the study. Children with syndromic CLP, unilateral CLP or without surgical repair of the lips and palate were excluded from the cleft group. All children with BCCLP and their parents expressed consent to voluntary participation in the examination. As a result of applying the adopted inclusion and exclusion criteria, 20 patients with cleft (10 girls and 10 boys) aged 6.6 to 12.5 years (mean age 9.6 ± 1.9) were enrolled in further studies. 

The control group consisted of 57 patients without CLP (34 girls and 23 boys) aged 6.8 to 11.5 years (mean age 9.2 ± 1.7). They were selected from among 120 children who were referred to the Orthodontics Clinic in Szczecin in 2017. Sixty-three children were excluded because either they did not express consent to participate voluntarily in the study (five patients) or did not meet other inclusion criteria (58 children). The inclusion criteria for the control group were as follows: subjects of both sexes with mixed dentition, children without malocclusion (Class I occlusion with a proper relationship between the upper and the lower dental arches), with normal lip seal, without speech disorders, with no prior orthodontic treatment and those who expressed consent to voluntary participation in the study. Participants with malocclusion, abnormal lip seal, speech disorders and those who had already finished their orthodontic treatment or were undergoing orthodontic treatment at the time of the investigation were excluded from the control group. 

All children were assessed by means of a clinical and electromyographic examination. In the first part of the examination the general medical records of the patients were analyzed. They provided some demographic and social data as well as information about all lip and palate operations the child had undergone, such as the cleft center, operating method, timing and sequence of surgical reconstructions, and details on the masticatory organ including the presence of speech disorders. Then, children were clinically investigated by a single assessor. The patients’ facial features were evaluated on the base of specific anthropometric landmarks. A clinical examination was also performed to assess the type of lip seal. When the lips are short and cannot close without effort, this is called “lip incompetence”. The intraoral examination consisted of an analysis of the shape of the dental arch on three planes together with a mutual analysis of the upper and lower arches. Table 1 presents the characteristics of the study groups. 

**Figure 1:**
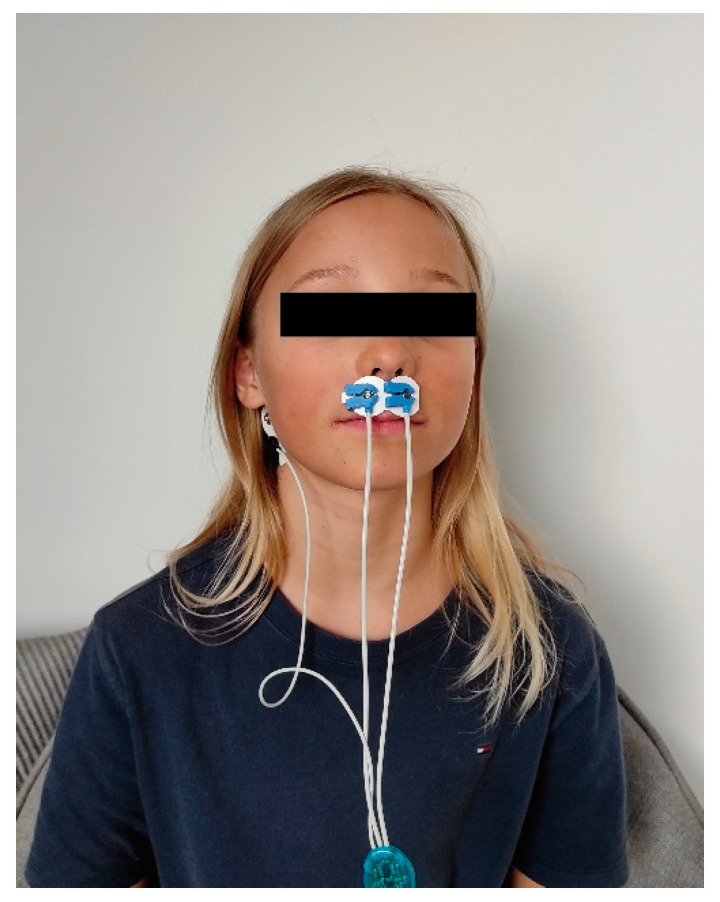
Location of surface electrodes (Noraxon Dual Electrode, Noraxon, USA) during EMG examination of the upper lip.

The next stage of the research was the EMG analysis of the superior orbicularis oris muscle. These methods were used by Szyszka-Sommerfeld et al. [23]. The EMG assessment required each patient to sit in a standard position in a dentist’s chair without a headrest. Each subject was asked to assume a natural head position that would avoid the undesirable tilt of the head [39]. The EMG recordings were made using a DAB-Bluetooth device (Zebris Medical GmbH, Isny im Allgäu, Germany) with a 1000× gain level, 7 Hz–5 kHz high-pass filter, 1 kHz channel sampling rate, 12 bit analogue-to-digital converter dynamic resolution range, and an input impedance for analogue channels of 146 kΩ. Disposable, self-adhesive silver/silver chloride (Ag/AgCl) bipolar surface electrodes (Noraxon Dual Electrode, Noraxon, Scottsdale, AZ, USA) were placed on the upper orbicularis oris muscle at a constant distance between the electrodes 20 mm along the line from the lip commissure to the nose (subnasal point) [22,23] and the reference electrode was placed lower and behind the right ear (Figure 1).

Prior to EMG recordings, the patient’s skin area of interest was cleaned with a 70% ethyl alcohol solution. After this cleaning had been carried out an impedance test was performed with a Metex P-10 measuring device (Metex Instruments Corporation, Seoul, Korea) to verify that the examined area had been correctly prepared (low skin tissue impedance). The condition for starting the electromyographic examination was skin impedance, ultimately 1 × 10^3^–30 × 10^3^ Ω. EMG recordings started 5 min later.

The electrical activity of the upper orbicularis oris muscle was measured by means of electromyography while the following situations were tested:

1. At rest with the lips relaxed.
2. Saliva swallowing.
3. Protrusion of the lips.
4. Compression of the lips.
5. Formation of the bilabial phoneme /p/ associated with the vowel /a/ in a consonant/vowel context.
6. Formation of the bilabial phoneme /b/ associated with the vowel /a/ in a consonant/vowel context.
7. Formation of the bilabial phoneme /m/ associated with the vowel /a/ in a consonant/vowel context.

For standardization purposes, all situations were considered movement, including resting with a relaxed mouth. To ascertain stability, all movements were repeated at least three times. The first EMG measurement was not taken into account since it was frequently noted that they varied significantly in relation to the other two repetitions. Thus, for one subject, all EMG data was the arithmetic mean of the last two EMG recordings. There was an interval of about 1 min between each action.

The DAB-Bluetooth instrument has been synchronized with the computer to process the data and display it graphically. After this operation had been performed the data was normalized in relation to the amplitude using the peak EMG value. This standardization procedure was a fundamental step in the preliminary processing of raw data to ensure further reliable analysis. The activity with higher values was taken as the maximum reference point for the muscle. The values of the protrusion of the lips of all subjects gave the highest average of all movements. Therefore, this situation served as a reference, representing 100% of the muscle activity of the upper orbicularis oris muscle. Finally, the normalization process involved expressing measured values as a percentage of the reference value, i.e., lip protrusion, according to the following formula: mean values of electrical activity (µV) during movement/mean values of electrical activity (µV) during lip protrusion x 100% [23]. All the EMG measurements were expressed as normalized values (unit µV/µV%).

The repeatability of the recording procedure was assessed on the basis of duplicated measurements of muscle electrical activity performed on 20 children by the same examin-er. There was a 15-min break between the first and second EMG examinations. The results are presented in Table 2.

The statistical analysis was performed using STATISTICA software (version 13). The Mann-Whitney U test was used to compare the EMG results between analyzed groups (Table 3 and Table 4) and the EMG measurements on repeatability of the recording protocol (Table 2). The significance level was set at *p* = 0.05. 

## 3. Results

The characteristics of the cleft and noncleft groups are presented in Table 1. Posterior crossbites were diagnosed in 70% of the subjects with BCCLP, while 65% of the children with CLP were diagnosed with lateral open bites. Class III malocclusions were diagnosed in 55% of the patients with clefts.

Table 2 shows the results of the repeatability of the electromyographical procedure. The differences of an upper lip activity were not statistically significant taking into ac-count the first and second EMG examination (*p* > 0.05).

The EMG data, i.e., the activity of the upper orbicularis oris muscle at rest and when swallowing saliva, activity during compression of the lip and the production of the bilabial phonemes /p/, /b/, and /m/ associated with the vowel /a/ for both groups, are presented in Table 3. 

Upper lip rest activity was similar both in subjects with clefts, and children without clefts (*p* > 0.05). An analysis of the EMG measurements showed no statistically significant differences in the EMG activity of the upper lip during the formation of bilabial phonemes /b/ and /m/ associated with the vowel /a/ between children with BCCLP and subjects without BCCLP (*p* > 0.05) (Table 3).

**Figure 2:**
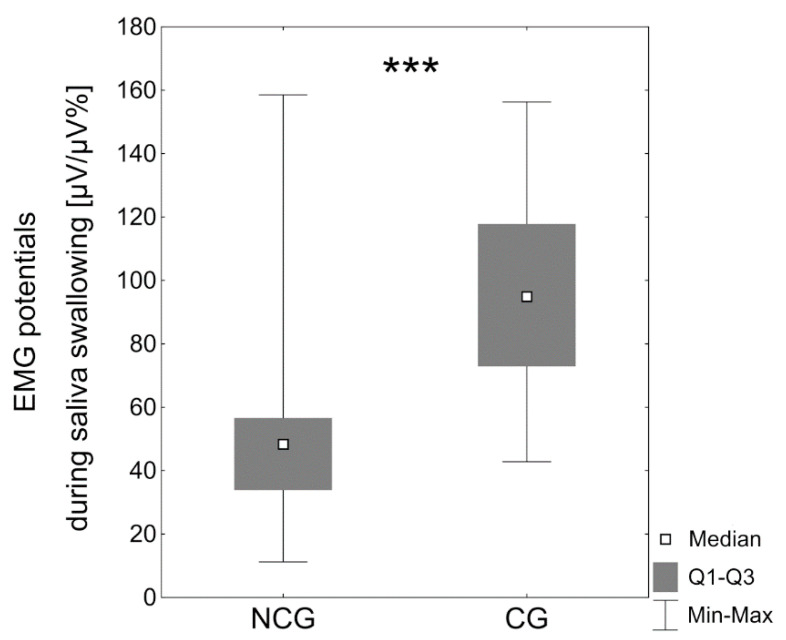
EMG potentials of the upper lip [µV/µV%] during saliva swallowing in the noncleft group (NCG) and the cleft group (CG); *** *p* < 0.0001.

During saliva swallowing, significantly higher median (Mdn) upper lip electrical activity was observed in patients with BCCLP (Mdn = 94.9 μV/μV%) compared to the noncleft group (Mdn = 48.3 μV/μV%, *p* < 0.0001) (Table 3, Figure 2). 

**Figure 3:**
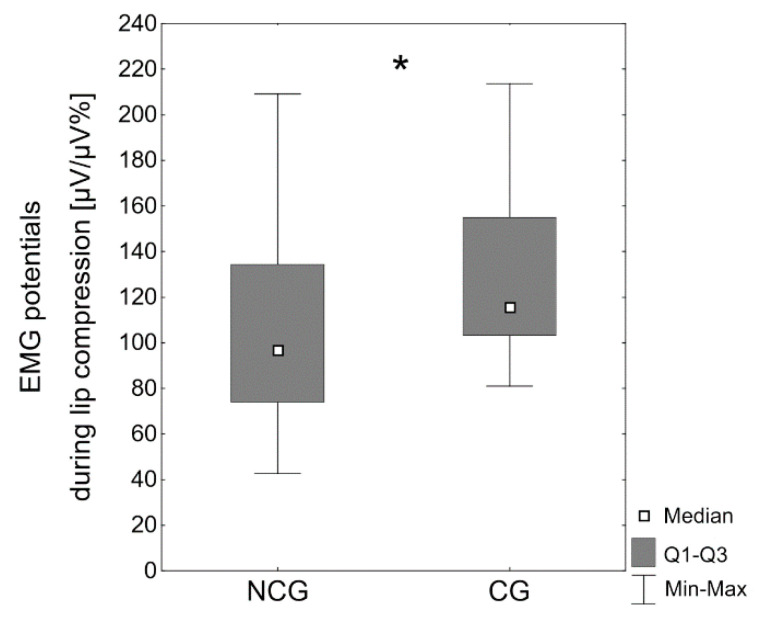
EMG potentials of the upper lip [µV/µV%] during lip compression in the noncleft group (NCG) and the cleft group (CG); * *p* < 0.05.

The EMG activity of the superior orbicularis oris muscle during lip compression was significantly higher in the cleft group (Mdn = 115.6 μV/μV%) compared with the subjects without clefts (Mdn = 96.9 μV/μV%, *p* < 0.05) (Table 3, Figure 3).

**Figure 4:**
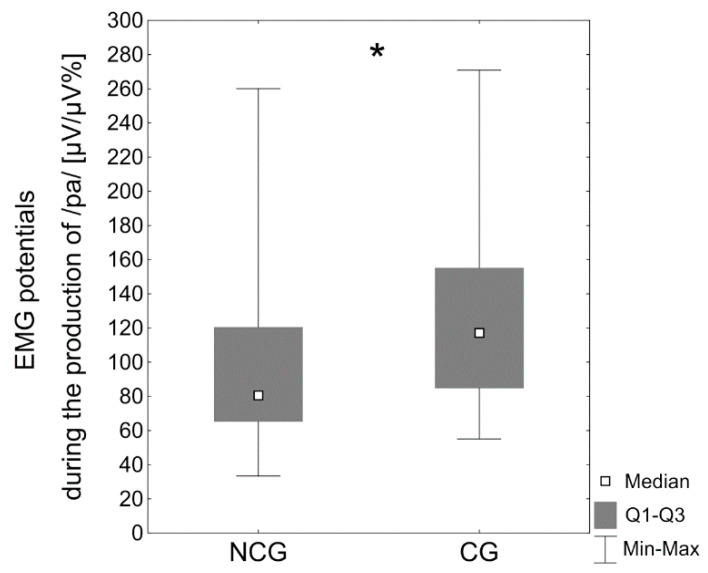
EMG potentials of the upper lip [µV/µV%] during production of the phoneme /p/ with the vowel /a/ in the noncleft group (NCG) and the cleft group (CG); * *p* < 0.05.

The median values for upper lip electrical activity were likewise considerably higher during the formation of the bilabial phoneme /p/ associated with the vowel /a/ in the children with BCCLP (Mdn = 117.2 μV/μV%) compared with the children with no cleft (Mdn = 80.6 μV/μV%, *p* < 0.05) (Table 3, Figure 4).

The values of the electrical activity of the upper lip within the BCCLP group depending on the lip seal (incompetent vs. competent), facial profile (concave vs. straight or convex), Angle class (class III vs. class I or class II occlusion), and overjet (negative vs. ≥0 mm) are presented in Table 4. During saliva swallowing, EMG potentials of the superior orbicularis oris muscle were significantly higher in patients with incompetent lip seal (*p* < 0.001), concave facial profile (*p* < 0.001), Class III malocclusion (*p* < 0.01), and negative overjet (*p* < 0.001) (Table 4).

## 4. Discussion

This is the first study to evaluate the electrical potentials of the upper orbicularis oris muscle in children who had been operated on for bilateral complete cleft lip and palate. Our analysis included only those surgically treated for bilateral CLP based on a similar operating protocol with a limited number of surgeons involved, thus ensuring sample homogeneity and reducing the number of confounders. The experiment showed increased EMG activity of the superior orbicularis oris muscle in children with BCCLP during saliva swallowing, lip compression and when producing the phoneme /p/ associated with the vowel /a/. As no similar studies on participants with bilateral CLP has been carried out it is difficult to compare our results with any others. Nevertheless, a few studies of lip electromyography in patients treated surgically for unilateral cleft lip and palate have been performed. In a recent study, Szyszka-Sommerfeld et al. [23] analyzed the electrical activity of the upper orbicularis oris muscle in patients aged 6 to 13 years. Their research cohort comprised 45 children operated on for unilateral complete CLP. These were compared to 40 control subjects. The data obtained in our study is consistent with the results reported by Szyszka-Sommerfeld et al., who found that the EMG activity of the upper orbicularis oris muscle while swallowing saliva and compressing the lips was significantly higher in the cleft group than in the patients with no CLP. In addition, the authors observed that upper lip EMG potentials both at rest and while speaking were comparable in both groups. They suggested that the increased activity of the repaired upper lip during saliva swallowing and lip compression in patients with unilateral complete CLP could affect facial morphology. Carvajal et al. [22] researched the function of the upper lip in children with CLP aged between 7 and 12. Children with CLP studied by Carvajal et al. with regard to cleft type constituted mixed sample (children with unilateral and bilateral CLP). There were no differences in the EMG potentials of the upper lip at rest between patients with and without clefts, while the EMG activity of the upper lip during saliva swallowing in children with clefts was significantly greater than in the subjects without CLP. The authors concluded that the upper orbicularis oris muscle exerts a limiting pressure on the growth of the jaw during swallowing. Genaro et al. [24] observed that the electrical potentials of the upper lip during speech and nonspeech movement tasks were higher in patients aged 15–23 with repaired unilateral cleft lips and/or palate compared with the subjects without clefts. The authors suggested that increased lip activity during function in patients with CLP may impact on their facial growth. Ravera et al. [38] measured the EMG activity of the upper lip in children aged 6 to 12 years surgically treated for unilateral cleft lip and palate. They found significantly higher scores for the electrical potentials of the upper orbicularis oris muscle at rest and when saliva swallowing in the cleft group, while electromyographic values during speech were similar in both subjects with clefts, and in children without a cleft. It should be noted that the similar levels we observed between children with BCCLP and in those without BCCLP regarding EMG activity of the upper orbicularis oris muscle at rest contradict the findings described by Ravera et al. This may be due to differences in the selection criteria of the studied groups. In other words, this study included children with normal and abnormal lip seal (as Szyszka-Sommerfeld et al. and Carvajal et al.), while Ravera et al. evaluated patients with clinically short upper lips and insufficient lip seal. 

Our research made use of surface electromyography (sEMG). The advantage of sEMG is its non-invasiveness, as it uses surface electrodes located on the skin surface, which is absolutely necessary in research on children [40]. It should be noted, however, that the use of surface electrodes in patients with clefts has some disadvantages, such as the ability to detect electrical potentials not only of underlying muscle activity, but also of surrounding connective tissue, skin thickness, and nearby nerves. This environment may have been different in these patients and may have accounted for the observed changes. When interpreting the study results it is also important to remember that patients with CLP had malocclusions. Moreover, 50% of children with BCCLP had incompetent lip seal. These factors may also contribute to variations in EMG pattern of the superior orbicularis oris muscle. In our study the electromyographical values of the upper orbicularis oris muscle during saliva swallowing were significantly greater in children with BCCLP and abnormal lip seal compared to the subjects with BCCLP and lip competence. Similarly, Carvajal et al. [22] stated that in a cleft lip and palate group, patients with insufficient lip seal produced the highest EMG potentials during saliva swallowing. What is more, Gustafsson and Ahlgren [41] demonstrated that EMG recordings of the upper lip in children with lip incompetence were significantly higher than in normal children when measuring lip closure, chewing, and swallowing.

It is known that abnormal craniofacial growth may contribute to severe occlusal dis-orders, which in turn could be a cause of muscle dysfunction [42]. Our findings suggest that the prevalence of malocclusions in patients with bilateral CLP is comparatively high. Many studies have focused on EMG analysis of muscles in the masticatory system in patients with malocclusion. It was confirmed that improper occlusion has a significant impact on muscle electrical activity [35,37,42,43,44]. For these reasons, it should be emphasized that altered EMG activity of the upper lip that was observed in children with repaired cleft may also be the result of the presence of malocclusions or abnormal lip seal.

For better understanding the upper lip EMG results during speech it is important to consider some details on speech disorders in patients with CLP. The major speech problems observed in children with cleft lip and palate include velopharyngeal inadequacy (VPI), hypernasality, nasal air emissions (audible and inaudible), weakness of production of high-pressure consonants, and compensatory articulation errors [45,46]. It should be noted that patients with CLP may present with articulation errors patterns as follows: developmental, obligatory and compensatory [46]. The children with developmental articulation errors they have problems producing sounds (articulation) and sound patterns (phonological processes) after the age of expected mastery. Obligatory articulation errors are produced given abnormal structures, causing the deformation of the sound. These errors include hypernasality, nasal emission and weak pressure consonants which can be associated with VPI, oronasal fistula and short utterances. Compensatory misarticulations refer to changes in placement of sounds to offset the anomalous structure. Usually, the child with repaired cleft is still trying to produce sounds using abnormal habits to “compensate” for previous structural deficiencies. The most prevalent compensatory articulation errors in patients with CLP include glottal stops, pharyngeal stops, pharyngeal fricatives, nasal fricatives, laryngeal fricatives, mid-dorsum palatal stops, and pharyngeal plosives [46,47]. In our study, children produced the bilabial phonemes /p/, /b/ and /m/ with the vowel /a/. These consonants differ according to the manner of articulation and pressure requirements. Thus, plosives sounds, such as /p/ and /b/ involve complete obstruction of the airstream followed by a rapid release that is heard as a plosion. This required a high air pressure in the oral mouth. As the intraoral pressure in subject with a cleft is usually insufficient patients with CLP and/or velopharyngeal dysfunction (VPD) have problems producing these consonants. The incapacity to create and/or preserve adequate amount of intraoral pressure for production of plosion may lead to changes in placement of generation this type of sounds, called compensatory articulation errors. In these articulation errors patterns, the manner of articulation is usually maintained, while the place of production is abnormally posteriorized. On the contrary, nasal sounds including /m/ are created with acoustic energy being displaced concurrently to oral and nasal cavities. These sounds do not need pressure build-up [47]. Based on the above considerations, it would be expected the reduction of the EMG potentials of the upper orbicularis oris muscle in patients with CLP during the production of the plosives sounds /p/ and /b/. On the other hand, it can be supposed that the EMG activity of the upper lip is higher in cases when the lips do not close in the rest position and increased scope of movement is necessary to achieve contact between the lips for the production of the bilabial phonemes /p/, /b/ and /m/. These situations may be the result of occlusal discrepancies, such as negative overjet. For these reasons, we must remember that our upper lip EMG results during speech movement tasks should be interpreted with caution. More additional studies in this area are needed to support and clarify these findings.

As mentioned earlier, it is significant that the increased electrical potentials of the upper orbicularis oris muscle in patients with BCCLP may indicate the greater force of contraction after surgical lip reconstruction to underlying structures [48], which may consequently affect maxillofacial growth. Particularly important seems to be the much greater electromyographic activity of the upper lip when swallowing saliva, because, although it is a very short-term activity, it is repeated up to 3000 times a day [1,49]. Previous studies [20,26,27,28,29] have shown that the restraining effect of increased labial pressure due to lip repair may disturb normal facial morphology by inhibiting maxillary sagittal growth in subjects with unilateral complete CLP. However, no such findings have been reported for patients with BCCLP. The most obvious cephalometric characteristic in adult patients with unoperated bilateral CLP is the protrusion of the premaxilla and prolabium, which results in excessive convexity of the midface and a significant shortening in the nasal columella [17,50]. The increased backpressure produced by the repaired orbicularis oris muscle can help correct the premaxillary protruding position, which results in an essential reduction in facial convexity. Such a long-lasting restraining effect on the projected premaxilla is usually beneficial, except when extreme lip pressure, an unfavorable growth pattern, or both retropositions the midface profile beyond the permissible sagittal limits [19,31,32]. In light of the above, it should be noted that the results of our study do not directly allow to conclude an association between EMG activity of the upper lip in patients with repaired clefts and maxillary growth and facial aesthetics. However, to support the hypothesis that the hyperactivity of the superior orbicularis oris muscle in children surgically treated for CLP may affect facial growth/aesthetics we analyzed electromyographic potentials of the upper lip during saliva swallowing within the BCCLP group. An analysis of the EMG values of the upper orbicularis oris muscle showed that the upper lip electrical activity in children with BCCLP and concave facial profile was significantly higher than in patients with straight or convex profile. Moreover, patients with CLP and class III malocclusion, as well as patients with negative overjet produced significantly greater EMG potentials during saliva swallowing. A further complementary study would be necessary to support and discuss these findings. In summary, an analysis of the function of the upper lip in growing patients undergoing surgery for cleft lip and palate may be significant for the clinical practitioners dealing with the multidisciplinary treatment of clefts and may become the basis for the development and the implementation of more effective methods of cleft therapy.

The limitations of the research, such as the small number of patients involved in cleft should be taken heed of, remembering that the results can only be considered a pilot study in this area. In addition, the study groups cover a relatively wide age range and some differences between patients may be due to discrepancies in their dentition and maturation of the neuromuscular system. The division of the study group into the smaller groups would allow the assessment of the degree of a progression or a regression of functional lip dysfunction in relation to the patient’s developmental age or would show a lack of such relationship. In this context, a further study would be necessary to confirm the study results. 

## 5. Conclusions

The electrical activity of the superior orbicularis oris muscle in children surgically treated for BCCLP was higher when swallowing saliva, compressing lips and producing the bilabial phoneme /p/ combined with the vowel /a/. This may be important for the clinical practitioners dealing with the multidisciplinary treatment of patients with clefts, especially in the aspect of the impact of surgical lip repair on the maxillofacial growth. It may become the basis for the improvement of methods of therapy in these patients. Further complementary studies would be necessary to confirm the study results.

## Figures and Tables

**Table 1:** The characteristics of the cleft and noncleft groups.

Variable		Cleft Group Mean Age 9.6 ± 1.9		Noncleft Group Mean Age 9.2 ± 1.7	
		*n*	%	*n*	%
Gender	Females	10	50	34	60
	Males	10	50	23	40
	Total	20	100	57	100
Place of residence	City	8	40	35	61
	Village	12	60	22	39
	Total	20	100	57	100
Speech disorders	No	2	10	57	100
	Yes	18	90	0	0
	Total	20	100	57	100
Lip seal	Competent	10	50	57	100
	Incompetent	10	50	0	0
	Total	20	100	57	100
Facial profile	Straight	7	35	50	88
	Concave	6	30	0	0
	Convex	7	35	7	12
	Total	20	100	57	100
Vertical overlap	≥0 <3 mm	5	25	57	100
	≥3 mm	6	30	0	0
	Reverse	9	45	0	0
	Total	20	100	57	100
Overjet	≥0 <3 mm	5	25	57	100
	≥3 mm	6	30	0	0
	Negative	9	45	0	0
	Total	20	100	57	100
Angle class	I	5	25	57	100
	II	4	20	0	0
	III	11	55	0	0
	Total	20	100	57	100
Posterior crossbite	No	2	10	57	100
	Unilateral	4	20	0	0
	Bilateral	14	70	0	0
	Total	20	100	57	100
Lateral open bite	No	7	35	57	100
	Yes	13	65	0	0
	Total	20	100	57	100

**Table 2:** EMG values of the study on repeatability of the recording protocol.

Activity	1 Examination					2 Examination				
	Min	Q1	Mdn	Q3	Max	Min	Q1	Mdn	Q3	Max
Rest	6.33	12.4	19.9	32.5	65.8	8.67	13.5	22.0	34.8	66.6
Swallowing of saliva	27.2	38.5	62.3	108.4	270.1	29.6	39.2	62.5	108.2	273.0
Compression of the lips	48.2	91.0	125.4	150.5	209.0	47.2	90.1	127.7	151.3	210.8
/pa/	33.1	57.0	98.8	134.2	265.8	33.2	58.71	103.0	136.5	263.9
/ba/	56.7	79.0	92.0	120.9	269.5	56.2	81.9	96.5	120.0	267.9
/ma/	34.6	66.1	92.6	155.0	260.0	34.1	65.5	96.7	156.9	262.5

Min: minimum, Q1: first quartile, Mdn: median, Q3: third quartile, Max: maximum. The Mann-Whitney U test.

**Table 3:** Electrical activity of the upper lip [µV/µV%] in the children examined in the study.

Activity	Cleft Group (CG)						Noncleft Group (NCG)					
	*n*	Min	Q1	Mdn	Q3	Max	*n*	Min	Q1	Mdn	Q3	Max
Rest	20	4.6	13.2	20.3	25.9	50.3	57	5.7	9.0	15.4	30.1	66.5
Swallowing of saliva	20	42.8	72.9	94.9	117.8	156.3	57	11.2	33.9	48.3	56.6	158.5
Compression of the lips	20	94.7	105.0	115.6	143.2	202.7	57	42.6	74.0	96.9	134.3	209.0
/pa/	20	55.0	84.8	117.2	155.1	270.9	57	33.4	65.3	80.6	120.4	260.1
/ba/	20	44.1	73.7	94.9	108.8	154.9	57	40.0	70.6	94.4	121.4	269.5
/ma/	20	27.8	56.9	88.0	139.2	248.7	57	30.5	61.0	85.4	120.9	167.7

Min: minimum, Q1: first quartile, Mdn: median, Q3: third quartile, Max: maximum. The Mann-Whitney U test.

**Table 4:** Electrical activity of the upper lip [µV/µV%] during saliva swallowing in the cleft group (CG).

Variable		*n*	Min	Q1	Mdn	Q3	Max
Lip seal	Competent	10	42.8	55.6	72.9	80.2	98.9
	Incompetent	10	93.7	99.3	117.8	127.5	156.3
Facial profile	Straight or convex	14	42.8	60.5	79.4	96.1	99.3
	Concave	6	111.7	123.9	127.5	137.5	156.3
Angle class	I or II	9	42.8	60.5	76.6	80.2	98.9
	III	11	51.7	96.1	111.7	127.5	156.3
Overjet	≥0 mm	11	42.8	55.6	76.6	91.1	98.9
	Negative	9	93.7	99.3	123.9	127.5	156.3

Min: minimum, Q1: first quartile, Mdn: median, Q3: third quartile, Max: maximum. The Mann-Whitney U test.

## Data Availability

The datasets used to support the conclusions of this article are included within the article. Access to other data will be considered by the corresponding author upon request.
